# Latest Developments in Artificial Intelligence and Machine Learning Models in General Pediatric Surgery

**DOI:** 10.1055/a-2689-8280

**Published:** 2025-09-05

**Authors:** Hesham Elsayed, Georg Singer, Tristan Till, Holger Till

**Affiliations:** 1 Department of Pediatric and Adolescent Surgery, Medical University of Graz, Graz, Austria; 2 Division of Pediatric Radiology, Department of Radiology, Medical University of Graz, Graz, Austria

**Keywords:** machine learning, artificial intelligence, pediatric surgery, review

## Abstract

**Introduction:**

Artificial intelligence (AI) and machine learning (ML) models rapidly transform health care with applications ranging from diagnostic image interpretation, predictive modeling, personalized treatment planning, real-time intraoperative guidance, and outcome prediction. However, their implementation in general pediatric surgery remains limited due to the rarity and complexity of pediatric surgical conditions, small and heterogeneous datasets, and a lack of formal AI training and competencies among pediatric surgeons.

**Materials and Methods:**

This narrative review explores the current landscape of AI and ML applications in general pediatric surgery, focusing on five key conditions: appendicitis, necrotizing enterocolitis, Hirschsprung's disease, congenital diaphragmatic hernia, and biliary atresia. For each, we summarize recent developments, including the use of AI in image analysis, diagnostic support, prediction of disease severity and outcome, postoperative monitoring, and histopathological evaluation. We also highlight novel tools such as explainable AI models, natural language processing, and wearable technologies.

**Results:**

Recent findings demonstrate promising diagnostic and prognostic capabilities across multiple conditions. However, most AI/ML models still require external validation and standardization. The review underscores the importance of collaborative, multicenter research based on joint datasets as well as targeted AI education for pediatric surgeons to fully explore the benefits of these technologies in clinical practice.

**Conclusion:**

AI and ML offer significant potential to improve pediatric surgical care, but broader implementation will require multicenter collaboration, a robust dataset, and targeted AI education for pediatric surgeons.

## Introduction

Artificial intelligence (AI) and machine learning (ML) are rapidly transforming health care, with applications ranging from diagnostic image interpretation, predictive modeling, personalized treatment planning, real-time intraoperative guidance, and outcome prediction.

While numerous studies have explored AI applications in adult medicine and pediatric radiology, the application of AI in general pediatric surgery remains in its early stages. 1 2 3 4 5 This is likely due to the rarity of many pediatric surgical conditions, which often require highly individualized approaches. Furthermore, the wide variability in patient age—from preterm infants to adolescents—as well as differences in size, anatomy, physiology, and disease presentation pose challenges for standardization. AI/ML models typically require large and relatively homogeneous datasets, making their application in pediatric surgery for rare diseases even more complex. 6 7 8

In pediatric AI research, datasets are often smaller, and less uniform compared with those in adult populations, limiting both the development and external validation of AI/ML models. These limitations underscore the need for AI systems that are interpretable, rigorously validated, unbiased, and specifically tailored to pediatric populations or, in some cases, even to individual patients. 6 9

Understanding the current level of AI knowledge and usage among pediatric surgeons is crucial for the effective implementation of these technologies in clinical practice in the future. Despite a growing interest, most pediatric surgeons have only basic knowledge of AI/ML and lack formal training. A recent survey amongst members of the European Society of Pediatric Endoscopic Surgeons found that 65% of respondents rated their AI/ML knowledge as basic, 86% had no formal training, yet 95% expressed interest in further education, particularly through workshops and hands-on learning. 10 Similarly, targeted workshops among pediatric surgeons in training have been shown to significantly improve participants' AI-related knowledge and confidence. 11 Other studies demonstrate that AI-assisted learning modules can enhance diagnostic accuracy and accelerate the learning curve in the evaluation of pediatric fractures. 12 13

**Fig. 1:**
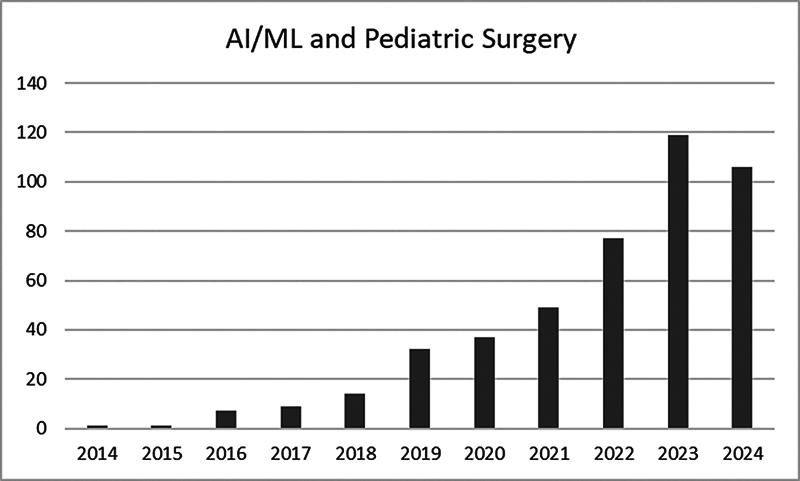
Number of publications found in PubMed with the search terms (“artificial intelligence” OR “machine learning”) AND “pediatric surgery” published between 2014 and 2025 as of July 10, 2025. AI, artificial intelligence; ML, machine learning.

Despite the before-mentioned challenges, the number of AI-related publications in pediatric surgery is increasing rapidly ( Fig. 1 ). To provide the best care, pediatric surgeons must stay informed about these rapidly evolving technologies.

This narrative review, therefore, aims to summarize the current literature on the applications of AI and ML in conditions relevant to pediatric surgery. To achieve this goal, PubMed was screened, and relevant articles were retrieved and summarized. Additionally, Table 1 provides an overview of the most commonly used AI and ML models in these studies, including their underlying principles, advantages, and limitations.

**Table 1:** Commonly applied artificial intelligence/machine learning models in pediatric surgery with their core principles, strengths, and limitations

Model	Published	Core principle	Strengths	Limitations
k-Nearest Neighbors	1952	Classifies items based on the k-closest data points	Simplicity, no training phase, high explainability	Data-sensitive, computationally expensive for large datasets
Logistic Regression	1958	Computes class probability using internal weights and a non-linear sigmoid function	Simplicity, explainability, and computationally inexpensive	Limited performance with complex, non-linear data
Ridge Regression	1970	Linear regression with an additional penalty term (L2 regularization) to reduce the complexity of internal weights	Simplicity, explainability, computationally inexpensive, and reduced risk of overfitting	Limited performance with complex, non-linear data, necessitates feature scaling, additional hyperparameters to tune
Decision Tree	1984/86	Build a tree of (binary) decisions to successively split data into distinct subgroups for classification	Simplicity, explainability, and interpretability	Limited generalization, error-prone on small datasets
Ensemble Voting Classifier	1990s	Aggregate predictions from multiple (potentially different) ML models by voting mechanism	Robust, high generalization, scalability	Complexity, low interpretability, requires additional design decisions
Support Vector Machine	1995	Finds optimal decision boundary (“hyperplane”) to separate data points of different classes by maximizing the distance between the boundary and the next closest point of each class	Simplicity, effectiveness on linearly separable datasets	Requires hyperparameter tuning, low scalability
Random Forest	2001	Train multiple decision trees on random subsets of data and features to use in an Ensemble Voting Classifier	Robust, good accuracy on large datasets, and interpretability	Computationally expensive on large datasets, requires hyperparameter tuning
Deep Neural Network	2012	Artificial neural network with multiple hidden layers between the input and output that learn progressively more complex, non-linear relations in the data	High flexibility, high capability of learning complex, non-linear relations	Low explainability and interpretability, reliance on large amounts of labeled data, computationally expensive to train
U-Net	2015	Deep neural network employing a symmetric structure with an encoder (input compression and feature extraction) and decoder (feature expansion and prediction)	High flexibility, high capability in particular for image-based applications	Low explainability and interpretability, reliance on large amounts of labeled data, computationally expensive to train
eXtreme Gradient Boosting	2016	Sequentially build decision trees that correct predecessors' error in a weighted ensemble for prediction.	High capabilities on structured (e.g., tabular) data, robust against outliers	Low explainability and interpretability, reliance on hyperparameter tuning

Abbreviation: ML, machine learning.

## Appendicitis

Acute appendicitis (AA) is the most common cause for emergency surgery in children and adolescents. 14 Early diagnosis is crucial as delayed identification increases the risk of perforation and its associated morbidities. 15 However, correct diagnosis is still challenging, and even scoring systems, including the Alvarado score and the Pediatric Appendicitis Score (PAS), are limited in their clinical impact. 16 17 No single history, physical examination, laboratory finding, or score can eliminate the need for imaging studies such as ultrasound or computed tomography. 17 However, ultrasound is user-dependent, therefore, limiting its diagnostic capacity, and CT, albeit having a high diagnostic accuracy, requires radiation exposure. 18 Taken together, it is not surprising that there is an increasing number of reports evaluating AI algorithms in the setting of pediatric AA.

By analyzing ultrasound images, ML models can improve diagnostic accuracy and reduce subjectivity, even in less-experienced users. Several studies have applied ML to ultrasound imaging to assist in the diagnosis of pediatric appendicitis. Marcinkevics et al demonstrated how interpretable ML models, that is, concept bottleneck models, based on ultrasound, can accurately detect AA in children, while also making the algorithm's reasoning more transparent for physicians. 19 20 Moreover, the same group investigated random forests, logistic regression (LR), and gradient boosting machines to predict diagnosis, management (surgical vs. conservative), and severity (complicated vs. uncomplicated) using data from 430 pediatric appendicitis cases (aged 0–18). The random forest model achieved strong performance with area under the precision–recall curve (AUPR) values of 0.94 for diagnosis, 0.92 for management, and 0.70 for severity. These findings were used to develop an online Appendicitis Prediction Tool for children with suspected appendicitis. 20

Similarly, in another study using several explainable AI techniques on a dataset consisting of 465 appendicitis cases and 317 non-appendicitis cases, an interpretable and transparent ML framework for rapid pediatric appendicitis diagnosis proposes a random forest model that integrated clinical and laboratory data to quick and safe diagnosis. Among others, the critical variables were appendix on ultrasonography and appendix diameter, supporting other reports that have shown that AI/ML models can be successfully used on pediatric ultrasound images in children suspected of appendicitis. 21 In another study, 50 videos and 6,914 images were used to train an AI neural network, showing that ML-assisted ultrasound evaluation improved sensitivity in diagnosing appendicitis in children while being transparent for clinical users, noting, however, that if failing to detect, examiners might be negatively affected. 22

To account for the more complex nature of pediatric appendicitis, a range of different AI models have been developed and tested that combine medical history, laboratory results, and physical examinations. 23 24 One key example is a random forest classifier trained on a wide range of clinical parameters. This model cannot only predict whether a child has AA, but also assess the severity and suggest the best treatment approach. 25 Other ML models have been developed to reduce unnecessary surgeries in children with suspected appendicitis. Trained on a dataset from 551 patients, the random forest model outperformed the appendicitis inflammatory response (AIR) score and achieved near-perfect sensitivity while potentially avoiding 17% of negative appendectomies. It also showed strong accuracy to differentiate complicated (gangrenous appendicitis) from uncomplicated ones (phlegmonous appendicitis), showing great potential for both diagnosing and treatment guidance. 16 In another study by Erman et al, an ML model was developed to also predict the severity of pediatric appendicitis across five grades using clinical and operative data from 1,980 children. The most promising model achieved 70.1% accuracy, offering a novel preoperative method to personalize treatment. 26 These advances suggest that AI models hold great promise in increasing diagnostic reliability while reducing the need for ionizing imaging methods like CT in children.

Another practical tool, the AI Pediatric Appendicitis Decision-tree (AiPAD), has been developed based on a model based on clinical and laboratory parameters without imaging data. AiPAD was highly accurate in predicting appendicitis and may offer a tool to be used in diagnosing appendicitis in children without the need for imaging. Nevertheless, the results of this study are based on a small number of patients. 27

Another innovative use of AI involves natural language processing (NLP). Instead of manually reviewing operative and ultrasound reports, NLP can now self-label these free-text documents automatically—making it easier to assess and track the severity of appendicitis. 25 Such efforts could make it easier to effectively analyze large datasets. Nevertheless, such AI solutions require further rigorous validation.

In addition, analysis of data derived from wearable technologies might be of value to predict postoperative complications. A study by Ghomrawi et al analyzed data from a consumer-grade wearable device recording multimodal data about daily physical activity, heart rate, and sleep, analyzed with a balanced random forest classifier in a cohort of children following appendectomy. 28 The ML model accurately detected 83% of abnormal recovery days in complicated appendicitis and 70% of abnormal recovery days in simple appendicitis prior to the true report of a symptom/complication. Such results could open new doors for monitoring children after surgery from the comfort of home and revolutionize hospitalization management.

In summary, AI and ML applications in pediatric appendicitis show promising results, offering improved diagnostic support, management guidance, and complication prediction for this common condition.

## Necrotizing Enterocolitis

Necrotizing enterocolitis (NEC) remains a devastating disease primarily affecting preterm neonates, with high morbidity and mortality. Early prediction, diagnosis, and effective management are of utmost importance and demand innovative solutions. 29 In the future, AI may contribute to the early prediction and diagnosis of this lethal gastrointestinal disease seen in neonatal intensive care units.

Recently, the performance of ML models using both birth characteristics and continuous vital signs from the first days after birth in the early prediction of NEC in preterm infants has been examined. 30 Verhoeven et al compared three ML models—including LR, support vector machine (SVM), and eXtreme gradient boosting (XGBoost)—reporting F1 scores ranging from 0.76 to 0.82 and AUPR values between 0.77 and 0.83. Notably, splanchnic and cerebral oxygenation were the strongest predictors. This study is especially relevant as a minimum gap of 48 hours was maintained between the last included data point and the clinical onset of NEC, emphasizing true prediction rather than diagnosis of early-onset cases. 30 In a nationwide Korean study, Kim et al used 38 variables—including maternal, prenatal, and postnatal factors obtained within 1 week of birth—to train an ML algorithm to predict surgical NEC, achieving an AUC of 0.721. 31 ML algorithms have also been applied to stool samples; in one study, longitudinal stool microbiota profiles from preterm infants at risk for NEC enabled prediction approximately 8 days before onset. 32

While NEC can be identifiable using ultrasound examinations, 33 abdominal radiographs remain the diagnostic gold standard. 34 Deep neural networks have been optimized to perform comparably to surgical residents in identifying pneumatosis on radiographic images. 35 Furthermore, deep learning models can achieve excellent diagnostic performance in predicting the need for surgical intervention based on abdominal X-rays. 36 Lure et al demonstrated that random forest and ridge logistic regression models can differentiate NEC from spontaneous intestinal perforation. 37

To enhance NEC diagnosis on radiographs, Lu and coworkers analyzed X-ray images from 484 patients (262 NEC patients and 222 non-NEC) and developed a radiomics model. 38 The combination of their radiomics models and radiologists in parallel diagnosis increased the sensitivity for diagnosing NEC and reduced the rate of missed diagnoses, highlighting AI's value as a diagnostic aid. Other studies using Gradient-weighted Class Activation Mapping have shown that the model's attention was focused on the fixed dilatation of the intestinal folds, intestinal wall edema, interintestinal gas, and portal venous gas on abdominal radiographs. 39

In patients suffering from surgical NEC, the choice between laparotomy (LAP) or comfort care (CC) represents a complex, ethical dilemma. In a recent retrospective study, Verhoeven and colleagues trained a behavioral artificial intelligence technology (BAIT) decision aid on expert knowledge. 40 BAIT is a novel technology that allows for developing decision aids. The authors have tested this technology on 40 patients (20 LAP and 20 CC) and have found that treatment choices by AI aligned with clinical practice in at least 80% of cases.

AI has already demonstrated promise as a supportive tool in the prediction and diagnosis of NEC. However, most of the datasets for training, validation, and testing are relatively small and often originate from a single institution. Given the complexity and rarity of NEC, larger prospective, multicenter studies are needed to fully evaluate the potential of AI in timely prediction, accurate diagnosis, and clinical decision-making. Additionally, external validation sets should be established to enable independent assessment of AI tools for NEC.

## Hirschsprung's Disease

Contrast enemas (CEs) are widely used as an initial diagnostic tool in the evaluation of patients with suspected Hirschsprung's disease (HD). However, their interpretation is known to vary significantly across institutions. 41 One of the first studies applying ML to quantitative imaging features extracted from barium enema for distinguishing HD from non-HD cases was published in 2021. 42 The analysis included 54 neonates with biopsy-confirmed short-segment HD and 59 neonates without HD. By adding five clinical features—including vomiting, abdominal distention, meconium extracted within 24 hours, and findings on rectal palpation—to the radiological features, SVM, and L2-regularized LR achieved an accuracy of 86.4%, a sensitivity of 81.8%, and a specificity of 90.9%. 42 More recently, a follow-up study evaluated the interobserver agreement and diagnostic performance of pediatric surgeons, pediatric radiologists, and a deep neural network in the interpretation of CE for HD. 43 The deep neural network achieved high diagnostic accuracy (area under the receiver operating characteristic curve (AUC-ROC = 0.87)), which further improved when combining anteroposterior and lateral views (AUC-ROC = 0.92). Integration of clinical data also enhanced sensitivity and negative predictive value. These studies underscore the potential of AI—particularly ML—in improving the diagnostic utility of CEs in HD.

The second diagnostic cornerstone in HD is rectal biopsy—which is either performed as rectal suction or full-thickness biopsy. Histologically, HD is characterized by aganglionosis and hypertrophic preganglionic nerve fibers with increased acetylcholine activity. In addition to standard hematoxylin–eosin (H&E) and acetylcholinesterase staining, various immunohistochemical markers—including S100, β-tubulin, glial fibrillary acidic protein, nerve growth factor receptor, and cell body markers such as calretinin, microtubule-associated protein 2 (MAP2), and peripherin—can aid in histopathologic assessment. 44 Most of these methods involve simplifying the detection of ganglionic cells or hypertrophic nerve fibers. These markers help simplify the detection of ganglion cells or hypertrophic nerves, yet histologic diagnosis still largely depends on expert interpretation. In 2019, Schilling et al demonstrated that by applying ML via an ensemble voting classifier, an accuracy of 87.5% could be achieved. Automated diagnosis using digital pathology with immunohistochemical panels showed promising results for markers like calretinin and MAP2. 44 Braun and colleagues confirmed these findings, showing that an AI-based analysis of parasympathetic hyperinnervation could identify HD with high precision. 45 Similarly, Greenberg et al developed an AI algorithm capable of accurately detecting ganglion cells in H&E-stained colon specimens. 46 Duci et al applied a U-net classifier to resected, fixed tissue samples and demonstrated reliable detection of ganglion cells and hypertrophic nerve fibers in HD specimens. 47 Trained on over 19,000 images, the models achieved accuracies of 92.3% and 91.5%, respectively. Another study revealed that a deep learning approach achieved accuracy values of over 90% in detecting ganglion cells in whole slide images of 366 frozen and 302 formalin-fixed paraffin-embedded H&E-stained slides obtained from 164 patients from three centers. 48 Notably, the use of heatmap overlays improved the diagnostic accuracy of pathologists from 77% to 85.8% and halved the average diagnostic time—from 139.7 to 70.5 seconds. 48 These developments highlight the potential of AI-based tools to assist in the histopathological diagnosis of HD and suggest a role in accelerating intraoperative frozen section analysis.

## Congenital Diaphragmatic Hernia

AI applications in congenital diaphragmatic hernia (CDH) are limited but emerging. Nevertheless, automatic segmentation models offer the potential to facilitate and standardize lung volume measurements, enhance data collection accuracy, and support the development of robust AI algorithms for predicting postnatal outcomes. 49 In 2024, Conte et al explored the feasibility of using a publicly available deep learning (DL)-based automatic segmentation system (nnUNet) for automatic MRI contouring of the lungs and liver of fetuses with CDH. 49 The performance of this system was compared with that of a human rater with 15 years of experience in fetal MRI. In a cohort of 39 CDH cases, the authors demonstrated that automatic segmentation of the fetal lung and liver is feasible and shows high accordance with manual segmentation. In a follow-up retrospective observational cohort study including 50 infants with isolated left-sided CDH, ML algorithms were evaluated for their ability to predict clinical outcomes. 50 The goal was to develop predictive models for mortality and persistent neonatal pulmonary hypertension based on integrated prenatal and early postnatal data. Three different classification algorithms were tested: XGBoost, SVM, and K-Nearest Neighbors (KNN). The best performing model, XGBoost, achieved 88% accuracy and 95% sensitivity for predicting mortality using 10 features and 82% accuracy for pulmonary hypertension severity with 14 features. The area under the ROC curve was 0.87 for mortality and 0.82 for pulmonary hypertension severity. 50

These promising results pave the way for further research into the use of AI and ML models to support clinical decision-making and outcome prediction in CDH patients.

## Biliary Atresia

Biliary atresia (BA) is a rare but severe neonatal cholangiopathy characterized by progressive fibroinflammatory obliteration of the extrahepatic bile ducts. If not detected or treated promptly, it can quickly result in liver cirrhosis and eventually liver failure. Early diagnosis and timely surgical intervention—typically via Kasai portoenterostomy—are critical for improving long-term outcomes and delaying the need for liver transplantation. 51 However, early identification remains challenging as clinical features such as persistent jaundice, acholic stools, and elevated liver enzymes are non-specific and can overlap with other causes of neonatal cholestasis.

AI-based diagnostic models have shown significant potential in improving the early and accurate identification of BA. Zhao et al developed a multimodal AI model incorporating serum biomarkers—most notably matrix metalloproteinase-7 (MMP-7)—along with additional laboratory and clinical variables, demonstrating excellent diagnostic performance. 52 In another study, ML techniques were applied to assess the diagnostic utility of gamma-glutamyl transferase (GGT) across various clinical settings, revealing that GGT's effectiveness is influenced by patient demographics. 52 Xu et al used bioinformatics and ML to analyze mRNA expression data and identified C-X-C motif chemokine ligand 8 (CXCL8) and thymosin beta-10 (TMSB10) as key diagnostic biomarkers for BA. CXCL8 has been implicated as a therapeutic target due to its proinflammatory role, while TMSB10 is associated with cell polarity, a factor potentially involved in disease progression. These findings were validated through immunohistochemistry and qRT-PCR, confirming upregulation of both genes in BA liver samples. 53

Ultrasound continues to be a cornerstone in diagnosing BA; however, its accuracy highly depends on the skill of the operator performing and interpreting the scan. Recent advances in deep learning have enabled automated analysis of ultrasound images with high diagnostic accuracy, aiding in the differentiation of BA from other causes of neonatal cholestasis. 54 These AI-driven tools offer consistent and reproducible results, which are particularly valuable in settings with limited access to experienced pediatric radiologists.

Beyond diagnosis, AI models have been utilized to stratify prognosis in BA. A study from China developed a novel, survival-based clustering model to classify BA patients into prognostically distinct subgroups, enabling early identification of candidates for liver transplantation. 55 This data-driven classification system provides a potential roadmap for personalized treatment planning and long-term management. In a related study, ML models trained on early clinical and laboratory data were developed to predict the likelihood of BA in infants presenting with cholestasis. These models demonstrated improved predictive accuracy and have the potential to reduce the reliance on invasive diagnostic procedures such as liver biopsy. 56

To improve generalizability, a multicenter cross-sectional study developed a robust ML-based diagnostic tool applicable across various clinical environments. 57 This approach highlights the scalability of AI-driven tools and their potential integration into routine clinical workflows. Moreover, predictive models have also been used to forecast postoperative complications such as adhesive small bowel obstruction following BA surgery, adding another layer of clinical utility. 58

## Conclusion

AI and ML hold immense potential to transform pediatric surgery, offering improvements in diagnostics, surgical precision, as well as personalized care. However, several challenges must be addressed, particularly within the pediatric context. These include the limited availability of large datasets due to the rarity of many pediatric surgical conditions. This fact underscores the need for data collection within international, multicenter collaboration. Furthermore, the heterogeneity of cases and variability in clinical practices across institutions require the development and optimization of robust AI/ML models. Additionally, deployment of such robust models should not be restricted to the developing institutions, but rather made available to anybody at any time and from anywhere via cloud hosting to support health and well-being anywhere and consequently reduce health care inequalities. On the other hand, ethical concerns represent another major hurdle—especially regarding decision-making involving neonates and children, where accountability and transparency are paramount. Finally, external validation of AI/ML models on independent datasets is essential to ensure their reliability and applicability in real-world settings. Collaborative, prospective data sharing will be key to refining and optimizing these promising technologies.

By understanding the fundamentals of these technologies and staying informed about their applications and developments, pediatric surgeons can harness their full potential to ultimately enhance patient outcomes.
